# Single view generalizable 3D reconstruction based on 3D Gaussian splatting

**DOI:** 10.1038/s41598-025-03200-7

**Published:** 2025-05-27

**Authors:** Kun Fang, Qinghui Zhang, Chenxia Wan, Pengtao Lv, Cheng Yuan

**Affiliations:** 1 College of Information Science and Engineering, Henan University of Technology, Zhengzhou, 450001 China; 2 Henan Center for Fair Competition Review Affairs, Zhengzhou, 450008 China

**Keywords:** Optical physics, Optics and photonics, Applied optics

## Abstract

3D Gaussian Splatting (3DGS) has become a significant research focus in recent years, particularly for 3D reconstruction and novel view synthesis under non-ideal conditions. Among these studies, tasks involving sparse input data have been further classified, with the most challenging scenario being the reconstruction of 3D structures and synthesis of novel views from a single input image. In this paper, we introduce SVG3D, a method for generalizable 3D reconstruction from a single view, based on 3DGS. We use a state-of-the-art monocular depth estimator to obtain depth maps of the scenes. These depth maps, along with the original scene images, are fed into a U-Net network, which predicts the parameters for 3D Gaussian ellipsoids corresponding to each pixel. Unlike previous work, we do not stratify the predicted 3D Gaussian ellipsoids but allow the network to learn the positioning autonomously. This design enables accurate geometric representation when rendered from the target camera view, significantly enhancing novel view synthesis accuracy. We trained our model on the RealEstate10K dataset and performed both quantitative and qualitative analysis on the test set. We compared single-view novel view 3D reconstruction methods across different 3D representation techniques, including methods based on Multi-Plane Image (MPI) representation, hybrid MPI and Neural Radiance Fields representation, and the current state-of-the-art methods using 3DGS representation for single-view novel view reconstruction. These comparisons substantiated the effectiveness and accuracy of our method. Additionally, to assess the generalizability of our network, we validated it across the NYU and KITTI datasets, and the results confirmed its robust cross-dataset generalization capability.

## Introduction

Methods for describing the 3D world in computing can be divided into two categories: 3D model reconstruction and 3D model generation. 3D model reconstruction varies widely depending on sensor types, with visual-based 3D reconstruction offering advantages such as low cost and rich texture information, making it indispensable in many fields. Visual 3D reconstruction has evolved alongside advancements in computer science and neural network algorithms, now encompassing two primary approaches: traditional methods and deep learning-based 3D reconstruction. In recent years, 3D model generation has also seen significant progress, with applications in game development, architectural design, and model evaluation. Currently, 3D model generation encompasses two types: text-to-3D^1–4^ model generation and image-based 3D model generation^5,6^. Whether for 3D model reconstruction or 3D model generation, a suitable 3D representation method is required to represent and store 3D data. Specifically, computer storage and representation of 3D scenes can be categorized into three types: explicit geometric representations (e.g., voxels, point clouds, triangular meshes, and depth maps); implicit geometric representations (e.g., neural radiance fields and signed distance fields); and hybrid geometric representations, such as Gaussian splatting. When using voxel grids to represent 3D scenes, the model’s precision is often insufficient for further processing and application. Increasing voxel grid resolution to improve accuracy, however, significantly increases storage requirements. The point cloud approach can only represent surface point sets of objects and lacks structure, with no topological connections between points. Triangle patches are convenient for rendering and can represent complex surfaces. However, as a non-structured network, obtaining them directly is challenging and requires intermediate calculations. Depth maps can only capture single-frame depth and are viewpoint-dependent. Although layered depth maps emerged to enable 3D scene representation and new viewpoint synthesis, this approach remains a 2.5D form of 3D representation. Due to the limitations of the explicit geometric representations outlined above, researchers have introduced implicit geometric representations, with NeRF^7^ and Signed Distance Function (SDF)^8^ being two of the most prominent methods.

Traditional visual-based 3D reconstruction methods, such as Structure-from-Motion (SfM) and Multi-View Stereo (MVS), rely on feature matching and triangulation to recover 3D geometry. These methods require multiple images from diverse viewpoints and often suffer from failure in texture-less regions, occlusions, or inaccurate matching. Moreover, they cannot synthesize novel views of the scene. In contrast, neural implicit representations like NeRF learn continuous volumetric scene representations directly from images and have shown remarkable performance in high-fidelity reconstruction and novel view synthesis. Implicit representations do not directly describe vertices, edges, or surfaces in space; instead, they define the relationships governing these spatial parameters. However, implicit representations also have several limitations, including limited editability and a less intuitive form of representation. 3DGS^9^ can be considered a fusion of explicit and implicit representation methods. It expands each point into an ellipsoid containing lighting information and renders images through rasterization operations. In order to address the inherent limitations of conventional 3D representation methods, recent research has increasingly turned to neural implicit scene modeling techniques, such as NeRF and 3DGS. These methods offer a compelling trade-off between rendering fidelity and learning efficiency, making them prominent directions in contemporary 3D vision research. However, both vanilla NeRF^7^ and vanilla 3DGS^9^ impose stringent requirements on reconstruction conditions, which limits the promotion and development of these new technologies. For instance, both vanilla NeRF and vanilla 3DGS have specific requirements regarding the number of input images, shooting angles, and consistency of lighting conditions. Additionally, these methods are trained and reconstructed on a per-scene basis, with little capacity for cross-scene generalization. In light of these limitations, researchers have proposed various improvement strategies to advance these new paradigms of 3D reconstruction. One promising direction of improvement is to achieve 3D scene reconstruction and novel view synthesis with a minimal number of input images^10^. The most challenging work in this direction involves reconstructing 3D scenes and synthesizing novel views from a single image. Monocular 3D reconstruction refers to the process of constructing a three-dimensional scene from a single image, enabling the synthesis of new views within a certain range of perspectives. This task is quite challenging. First, the reliance on a single image limits the geometric constraints that can be derived from multiple viewpoints. Additionally, to achieve the capability of new view synthesis, an appropriate three-dimensional representation must be chosen to construct the scene. Early works aimed to estimate scene depth using monocular depth estimation, subsequently employing point clouds for 3D reconstruction, which placed higher demands on the accuracy and generalization of monocular depth estimators. Meeting these requirements enables successful monocular 3D reconstruction from a single view. Despite significant advancements in deep learning-based monocular depth estimation, several challenges remain. Improving the generalization of monocular depth estimators necessitates a large training dataset; however, obtaining such precise datasets is challenging and costly. Consequently, researchers have turned to collecting large-scale relative depth datasets to enhance the cross-dataset generalization capabilities of neural networks. While addressing generalization issues has mitigated some challenges, training relative depth estimation models often neglects 3D geometric information, focusing solely on pixel-level regression. This oversight leads to unknown shifts in depth that cause shape distortions, rendering direct 3D reconstruction from depth estimation results infeasible. Subsequent research efforts have focused on improving geometric quality. Some studies suggest that unknown shifts in depth and inaccurate focal lengths can lead to significant shape distortions, making the recovery of these parameters crucial^11,12^. Other studies propose that canonical space transformation can resolve the metric ambiguity caused by focal length; they suggest transforming all training data to a canonical space so that all data can be roughly considered as captured by the same camera^13^. These methods effectively address the inaccuracies in geometric estimation from depth data. However, they primarily express three-dimensional scenes using point clouds, achieving only the reconstruction of surface-near point clouds without the capability for new view synthesis. The emergence of novel 3D representation methods like NeRF^7^ and 3DGS^9^ has effectively addressed the challenges mentioned above.

To tackle these challenges, we propose a 3D reconstruction framework that leverages the strengths of 3DGS for efficient and generalizable single-view scene modeling. In our work, we employ 3DGS to represent 3D scenes. A U-Net^14,15^ architecture is used at the front end to predict the position and parameters of a series of 3DGS spheres corresponding to each pixel. Then, we apply the vanilla 3DGS^9^ rendering strategy to rasterize the scene from the appropriate viewpoints. We trained our neural network on the RealEstate10K^16^ dataset and evaluated its generalization capability on the KITTI^17^and NYU^18^ datasets. Our method introduces four key innovations:

1. We remove the need for layered Gaussian structures used in prior works such as Flash3D, and instead allow the network to directly regress the full 3D Gaussian parameters (position, scale, rotation) for each pixel, resulting in a more compact and geometrically faithful representation.
2. We introduce a homography-based geometric consistency loss that leverages known camera poses to enforce view-aligned depth constraints, improving occlusion modeling and reducing overfitting to visible surfaces.
3. We design a patch-wise depth regularization loss based on Pearson correlation to enhance the local continuity of the predicted depth and improve robustness to depth noise.
4. Our method features a lightweight architecture that requires fewer computational resources and demonstrates strong generalization performance across RealEstate10K, KITTI, and NYU datasets.

Overall, our model demonstrates more efficient GPU usage during training and achieves superior reconstruction quality in both quantitative and qualitative assessments compared to existing single-view 3D reconstruction approaches.

## Related work

Early studies on single-view 3D reconstruction used point clouds to enhance geometric reconstruction quality by refining relative depth estimates, ultimately generating the corresponding 3D point cloud data.

However, this approach lacks the ability to synthesize novel views, and the limitations of using point clouds as a 3D representation constrain its further development and application. Recent work has explored 3D reconstruction and novel view synthesis using MPI^16,19–22^. One study by Google introduced a technique called "Stereo Magnification," which employs deep neural networks to learn and generate MPI representations from stereo camera images, producing enhanced stereoscopic images^16^. Training this neural network requires numerous pairs of static stereoscopic images. The authors identified YouTube real estate showcase videos as a valuable source, extracting and processing segments to create a substantial training dataset, resulting in the RealEstate10K dataset. Two years later, Google introduced another method enabling neural networks to predict MPI representations from single images^21^. By training on extensive datasets, this model achieves generalization and enables novel view synthesis. However, MPI consists of multiple RGB-alpha planes, each representing scene content at a specific depth. The primary limitation of MPI is that depth is fixed and discrete, constraining its 3D spatial representation capabilities. The ByteDance Vision Technology team integrated NeRF and MPI to propose a new 3D representation approach called MINE^19^. MINE enables 3D reconstruction, novel view synthesis, and depth estimation from a single image. The MINE network still has several limitations, such as a high sensitivity to noise or partially occluded data, which results in lower reconstruction accuracy in cluttered or dynamic environments. Additionally, the network’s high computational cost and substantial memory requirements may limit its scalability for real-time applications or large-scale tasks.

Recently, diffusion models have achieved remarkable success in generative tasks and have significantly advanced the field of single-view and sparse-view 3D reconstruction.

The latest works represented by DreamFusion^1^ and Magic3D^2^ have demonstrated the strong potential of diffusion models combined with NeRF for single-image or text-driven 3D generation. Additionally, significant progress has been made by combining diffusion models with various 3D representations beyond text-driven generation. Point-E^4^ utilizes point cloud representations to transform 2D images or pure text descriptions into high-quality 3D point clouds. GET3D^23^ integrates diffusion models with mesh representations to generate 3D meshes exhibiting complex topologies, detailed geometries, and high-fidelity textures. One-2-3-45^24^ builds upon Zero-1-to-3^25^ using NeRF networks for 3D model generation; however, this approach lacks directness and suffers from multi-view inconsistencies in the generated models. DiffRF^26^ is the first approach to directly generate 3D radiance fields in an end-to-end manner, but this work remains focused on generating single 3D objects rather than complete 3D scenes. Subsequent works, such as SSDNeRF^27^, ZeroRF^28^, and LRM^29^, similarly concentrate on single-object generation. MVSplat360^30^ integrates 3DGS to synthesize novel 360-degree views under sparse input conditions. DiffusionGS introduces a novel single-stage 3D diffusion model capable of generating objects and scenes from a single view, achieving notable performance in novel view synthesis of scenes. In addition to the advancements in diffusion models for 3D generation described above, significant progress has also been achieved in novel view synthesis under sparse-view conditions using NeRF and 3DGS. Both pixelNeRF^10^ and MVSNeRF^31^ leverage pre-trained networks to incorporate priors. In the original NeRF dataset, images are used solely for supervised training; however, in pixelNeRF, they serve both as input data and as supervision signals. This approach of fully utilizing known data enables pixelNeRF to achieve good results even under sparse input conditions. MVSNeRF also employs a pre-trained network to address the challenge of Few-Shot learning.

While pixelNeRF utilizes straightforward multi-view projection and averaging, MVSNeRF employs a relatively more complex MVS-base approach. However, the underlying principles and implementations of the two are highly similar. IBRNet combines classic IBR techniques^32^ with volumetric rendering to create a generalized IBR module capable of rendering high-quality views without per-scene optimization. DietNeRF utilizes a CLIP^33^-trained ViT^34^ to predict the semantic information of the Reference and Unknown Views, aligning them to prevent degeneration. This work is arguably the first to combine NeRF with textual information, inspiring and driving subsequent AIGC efforts such as Text-to-3D with NeRF^1,27,35–37^. FreeNeRF^38^ introduces two regularization strategies. The first is an annealing strategy for Encoding, which gradually activates higher-frequency components during training. This is referred to by the authors as Frequency Regularization. The second strategy is Occlusion Regularization, which penalizes the density field near the camera, thereby reducing artifacts like floating objects in NeRF. RegNeRF^39^ introduces optimization strategies for unknown viewpoints through Appearance Regularization and Geometry Regularization. Additionally, it employs an annealing sampling strategy, similar to FreeNeRF, to prevent the network from focusing on high-frequency features of an object when only a limited number of input images are available, which can lead to incomplete low-frequency learning and the emergence of “floaters.”

Synthesizing novel views from sparse inputs presents significant challenges for 3DGS as well. Several works have made significant advancements in this area of research. PixelSplat^40^ follows the approach of pixelNeRF, leveraging the efficient training and rendering capabilities of 3D Gaussian representations to achieve notable results. MVSplat^41^ introduces an efficient feed-forward 3D reconstruction model that predicts 3D Gaussian distributions from sparse multi-view images without requiring depth map estimation. Compared to PixelSplat, MVSplat reduces parameters by tenfold and doubles inference speed, while also improving rendering quality in appearance and geometry, with enhanced cross-dataset generalization. SparseGS^42^ proposes a method for training a coherent 3DGS 360-degree scene radiance field from sparse training views. This work combines depth priors with generative and explicit constraints to reduce background collapse, eliminate floating noise, and improve consistency for novel views. DNGaussian^43^ identifies that scene geometry degradation is primarily influenced by the initial positioning of Gaussian ellipsoids, which can be mitigated through depth constraints. Consequently, this work introduces hard and soft depth regularization to recover accurate scene geometry under coarse monocular depth supervision while preserving fine-grained color appearance. LM-Gaussian^44^ aims to generate high-quality 360-degree novel views from a limited number of input images by integrating multiple large model priors through four key modules. In its depth-aware initialization, it extends DUSt3R^45^ to estimate camera poses and detailed 3D point clouds. By incorporating depth priors and point cloud denoising, high-quality point clouds are generated for Gaussian initialization. In the multimodal regularization Gaussian reconstruction module, depth, normals, and virtual view constraints are incorporated to regularize the optimization process. In the iterative Gaussian refinement module, image diffusion priors enhance the 3DGS-rendered images, which then further refine the 3DGS optimization. Iterative introduction of diffusion model priors improves the detail and quality of novel view synthesis. The final module, scene enhancement, employs not only image diffusion priors but also video diffusion priors, further enhancing the realistic visual effects of 3DGS-rendered images.

In the aforementioned sparse-view 3DGS reconstruction methods, multiple views are required, with a minimum of two views needed to operate effectively. The works most similar to ours are two recent projects from the VGG team at Oxford University, specifically “Splatter Image”^46^ and “Flash3D”^47^. The former primarily focuses on 3D reconstruction of individual objects, while the latter, like our work, focuses on 3D reconstruction of entire scenes. The difference between our work and previous studies is that firstly, we did not enforce a mandatory stratification of the 3D Gaussian ellipsoid estimated by the UNet; instead, we allowed the neural network to predict the 3D Gaussian ellipsoid’s position based on the observed scene.

Secondly, to prevent the network from overfitting by learning an overspecific scene and thereby reducing the performance of new viewpoint synthesis, we constructed a homography depth loss function for the source and target views to regularize the training process.

Finally, to eliminate the effect of outliers in depth estimation on the network and to allow the network to extract useful information from the depth distribution of surrounding pixels and apply it to parameter estimation, we constructed a regularization loss function based on the Pearson correlation coefficient on the target view.

## Method

**Fig. 1:**
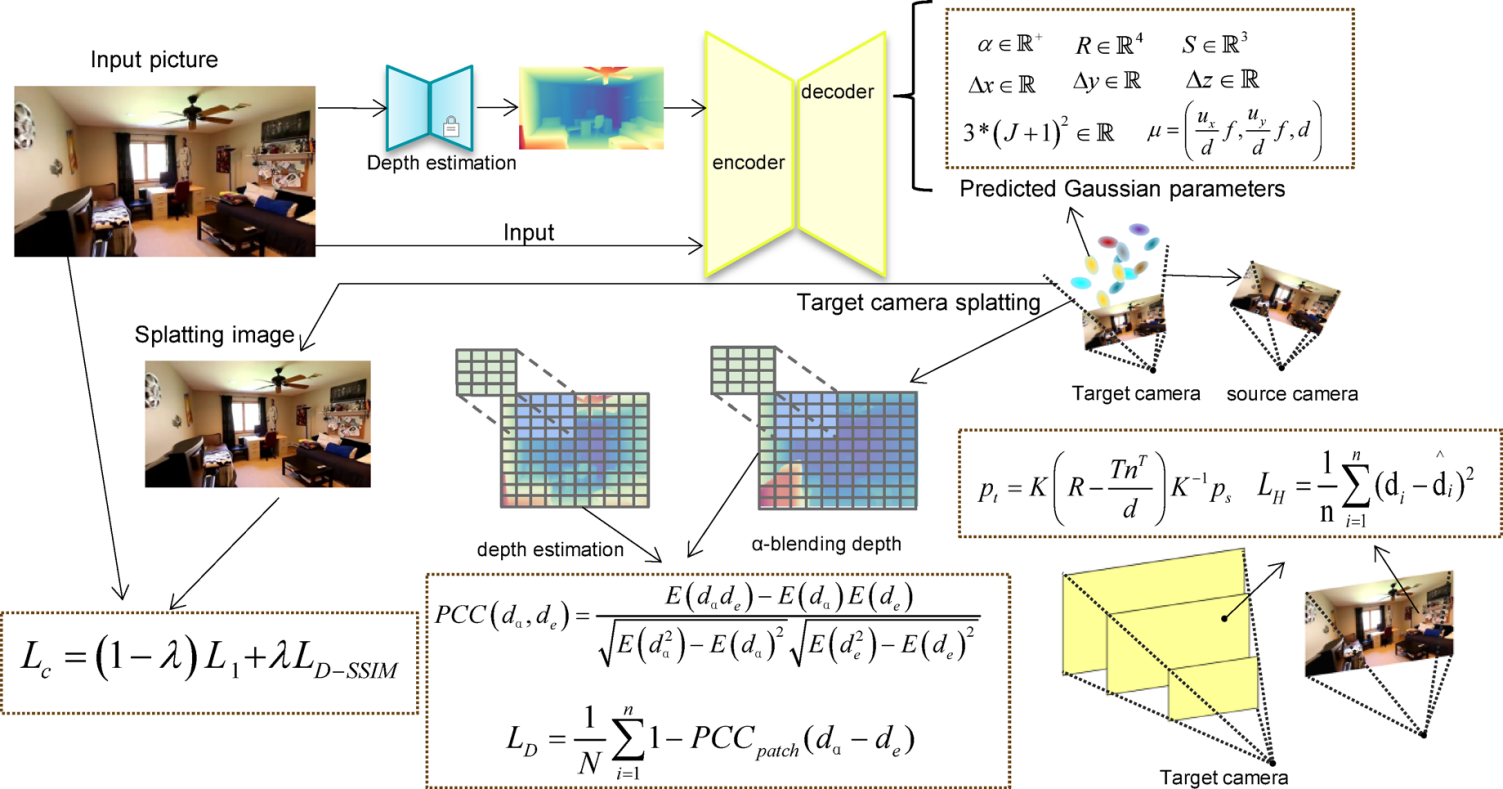
Overall Network Architecture. The input to the U-Net model includes both RGB images and depth values estimated by a monocular depth estimator. After passing through the encoder and decoder, the network predicts a set of parameters for each pixel, describing the corresponding 3D Gaussian ellipsoids and their offsets in the x, y, and z directions. To prevent the 3D Gaussian ellipsoids from overfitting to the depth surfaces of objects, which could lead to visually satisfactory renderings from the input view but inaccuracies from new perspectives, a homography depth loss function, denoted as L_H_, is defined based on the source camera parameters to regularize the Gaussian ellipsoid positions throughout network training. Additionally, a depth loss function is defined for the target camera rendering results. Instead of computing L2 loss on the depth values of corresponding pixels, the image is divided into patches of varying sizes, and the depth consistency is enforced by applying a Pearson correlation constraint on the depth information within each patch. The photometric loss for the rendered model continues to utilize the L1 loss and SSIM loss as applied in the vanilla 3DGS approach.

In this section, we will provide a detailed explanation of the implementation of our network. The overall architecture is shown in Fig. 1. The network takes a single image as input to achieve 3D scene reconstruction and novel view synthesis. Section "The principles of 3D Gaussian splatting" provides a discussion on the principles of 3DGS and the parameters constituting each Gaussian sphere. Finally, section "Single view generalization 3D Gaussian Splatting (SVG3D)" discusses our proposed method “Single view Generalizable 3D reconstruction based on 3D Gaussian splatting” (SVG3D).

### The principles of 3D Gaussian splatting

The vanilla 3DGS require sparse point clouds generated by the Structure-from-Motion^48^ (SFM) algorithm to initialize the positions of the 3D Gaussian spheres. The expression for each 3DGS sphere is given by:

1 $${G}_{i}\left(x,{\mu }_{i},{\Sigma }_{i}\right)=\frac{1}{\sqrt{{\left(2\pi \right)}^{k}\left|{\Sigma }_{i}\right|}}\mathit{exp}\left(-\frac{1}{2}{\left(x-{\mu }_{i}\right)}^{T}{{\Sigma }_{i}}^{-1}\left(x-{\mu }_{i}\right)\right)$$

where $x\in {\mathbb{R}}^{k}$ is a point in the *k*-dimensional image space (e.g., a pixel position), ${\mu }_{i}\in {\mathbb{R}}^{k}$ is the mean vector representing the center of the *i*-th Gaussian, ${\Sigma }_{i}\in {\mathbb{R}}^{k\times k}$ is the covariance matrix defining the shape and orientation of the Gaussian in screen space, Each 3D Gaussian sphere is defined by parameters that include the position coordinates μ, opacity α, covariance matrix Σ^49^, and spherical harmonics coefficients for RGB values in each Gaussian distribution. In practical applications, the coefficients in the expression can be simplified to facilitate computation. Thus, the vanilla 3DGS model uses a simplified formula to characterize each Gaussian sphere.

2 $${G}_{i}\left(x,{\mu }_{i},{\Sigma }_{i}\right)=\mathit{exp}\left(-\frac{1}{2}{\left(x-{\mu }_{i}\right)}^{T}{{\Sigma }_{i}}^{-1}\left(x-{\mu }_{i}\right)\right)$$

Each parameter dimension includes the Gaussian sphere center position $\mu \in {\mathbb{R}}^{3}$ and the opacity $\alpha \in {\mathbb{R}}^{+}$ at the center of the Gaussian sphere. Since any Gaussian sphere shape can be viewed as a standard 3D Gaussian distribution transformed through an affine transformation, this transformation impacts the covariance matrix through a rotation $R\in {\mathbb{R}}^{3\times 3}$ and a scaling $S\in {\mathbb{R}}^{3\times 3}$.The covariance matrix can thus be represented as

3 $$\Sigma =R\cdot S\cdot I\cdot {\left(R\cdot S\right)}^{T}=R\cdot S\cdot I\cdot {S}^{T}\cdot {R}^{T}$$

where $\Sigma \in {\mathbb{R}}^{3\times 3}$ is the 3D covariance matrix of the Gaussian, $R\in {\mathbb{R}}^{3\times 3}$ is the rotation matrix representing the orientation of the Gaussian, $S\in {\mathbb{R}}^{3\times 3}$ is the diagonal scaling matrix representing the axis-aligned standard deviations along the local x, y, z axes, $I\in {\mathbb{R}}^{3\times 3}$ is the identity matrix. To represent the anisotropy of each 3D Gaussian sphere’s color, spherical harmonics are employed to describe the color distribution at various points on each sphere. Finally, rendering the image is achieved through the principle of α-blending. In computer graphics and image processing, α-blending primarily addresses the issue of layer compositing. This process involves overlaying layers on a background to compute the final color, using each layer’s transparency β value as a parameter. Assuming that three images (I_1_ ~ I_3_) need to be blended onto a background image I_BK_, the fusion formula is represented as follows:

4 $${I}_{result}={I}_{1}\times {\beta }_{1}+\left(1-{\beta }_{1}\right)\left({I}_{2}\times {\beta }_{2}+\left(1-{\beta }_{2}\right)\left({I}_{3}\times {\beta }_{3}+{I}_{BK}\times \left(1-{\beta }_{3}\right)\right)\right)$$

**Fig. 2:**
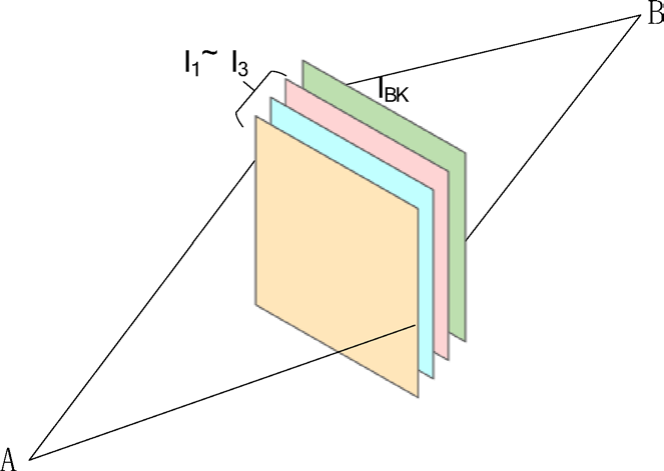
Transparency and Opacity Conversion 3DGS employs an alpha-blending technique that directly projects onto the observer’s viewpoint, differing from the conventional method in computer graphics where layers are rendered directly onto a background image. 3DGS is analogous to observing the image from point B, while background-based rendering corresponds to observing from point A. This difference results in identical mathematical expressions, but with a reversal in the concepts of transparency and opacity within the formulas.

After simplification, the formula becomes:

5 $${I}_{result}={I}_{1}\times {\beta }_{1}+\left(1-{\beta }_{1}\right){\beta }_{2}{I}_{2}+\left(1-{\beta }_{1}\right)\left(1-{\beta }_{2}\right){\beta }_{3}{I}_{3}+\left(1-{\beta }_{1}\right)\left(1-{\beta }_{2}\right)\left(1-{\beta }_{3}\right){I}_{BK}$$

where *I*_result​_ is the final rendered pixel color, *I*_1_, *I*_2_ ,*I*_3_ are the RGB colors contributed by the first, second, and third Gaussian (or layer), respectively, β1, β2, β3 ∈ [0,1] are the blending weights (or opacities) of each corresponding Gaussian, *I*_BK_ is the background color, typically used when no Gaussian fully occupies the pixel. In practical applications, the background color is typically ignored, meaning that the background pixel color can be assumed to be zero. By using mathematical induction, the formula for calculating each pixel when N images are blended is derived as follows:

6 $${I}_{result}={\sum }_{i\in N}{I}_{i}{\beta }_{i}{\prod }_{j=1}^{i-1}\left(1-{\beta }_{j}\right)$$

where the value β represents the transparency of the current layer. However, As shown in Fig. 2 Transparency and Opacity Conversion 3DGS employs an alpha-blending technique that directly projects onto the observer’s viewpoint, differing from the conventional method in computer graphics where layers are rendered directly onto a background image. 3DGS uses a forward projection method towards the viewpoint, rendering the image as if observed from viewpoint B.

In 3DGS, 3D Gaussian spheres are sorted and projected based on their depth. The final formula is consistent with the α-blending formula for layers, except that transparency β is replaced by opacity α, as shown in formula:

7 $$C={\sum }_{i\in N}{c}_{i}{\alpha }_{i}{\prod }_{j=1}^{i-1}\left(1-{\alpha }_{i}\right)$$

where *C* is the final pixel color after compositing all Gaussian contributions, *N* is the set of indices of all Gaussians projected onto the pixel, sorted in front-to-back depth order, *c*_*i*_ is the RGB color of the *i*-th Gaussian, α_*i*_ ∈ [0,1] is the opacity of the *i*-th Gaussian (i.e., how much it contributes to the pixel), ${\prod }_{j=1}^{i-1}\left(1-{\alpha }_{j}\right)$ accumulates the transparency of all Gaussians in front of the *i*-th one, ensuring that earlier (closer) Gaussians occlude later (farther) ones. The color of a 3D Gaussian sphere is represented by spherical harmonic functions, which can model the illumination at any given point in space. The formula for the lighting function is represented as

8 $$C\left(\theta ,\varphi \right)={\sum }_{j=0}^{J}{\sum }_{m=-j}^{j}{c}_{j}^{m}{Y}_{j}^{m}\left(\theta ,\varphi \right)$$

where *C*(θ,φ) is the directional color (or radiance) of a 3D Gaussian along the direction defined by spherical angles (θ,φ), θ ∈ [0,π] is the elevation angle (measured from the positive z-axis), φ ∈ [0,2π) is the azimuth angle (measured in the x–y plane), J is the maximum degree (or order) of the spherical harmonics expansion, controlling the level of detail, j is the degree index of the spherical harmonics basis function, m is the order index for a given degree *j*, with m ∈ [− j,j], ${c}_{j}^{m}$ is the learned spherical harmonics coefficient for the *j*-th degree and *m*-th order, representing the contribution of each basis function to the overall lighting, ${Y}_{j}^{m}\left(\theta ,\varphi \right)$ is the real-valued spherical harmonic basis function of degree *j* and order *m*, evaluated at direction (θ,φ).

The directional color of each Gaussian is modeled using spherical harmonics, allowing compact and rotation-aware encoding of view-dependent color effects. The coefficients ${c}_{j}^{m}$ are learned per-Gaussian, and the basis functions ${Y}_{j}^{m}\left(\theta ,\varphi \right)$ encode the angular structure of the illumination. Using a neural network, we predict the coefficients corresponding ${c}_{j}^{m}$ to each basis function to ultimately determine the color values of the Gaussian sphere from different observation directions.

### Single view generalization 3D Gaussian splatting (SVG3D)

Our objective is to train a neural network that can reconstruct 3D scenes from a single view and enable novel view synthesis within a certain range. This requires the network to exhibit both generalization and accuracy in 3D reconstruction. To achieve this, a basic approach would involve using a depth estimator to determine the central coordinates of 3D Gaussian spheres for each pixel, followed by splatting operations based on the positions and other parameters of these Gaussians to render images from various viewpoints. However, performing absolute depth estimation from a single image is quite challenging, and absolute depth estimators typically have poor generalization. In recent years, relative depth estimators based on deep learning have developed rapidly and found extensive applications in fields like augmented reality (AR), virtual reality (VR), robotics, and autonomous driving. Notable works in this area include DepthAnything^50^ and DepthAnythingV2^51^, which demonstrate strong cross-dataset generalization capabilities. However, relative depth estimators only account for the relative depth information between points, without considering the 3D geometric relationships among points on the object. Thus, using a relative depth estimator to directly predict the center positions of 3D Gaussian spheres is unfeasible. Furthermore, if we only predict 3D Gaussian Spheres constrained to depth surfaces, the model’s ability for novel view synthesis will suffer significantly. Therefore, the network must predict multiple 3DGS for each pixel to enable effective novel view synthesis. Another challenge is that the adaptive density control strategy in 3DGS involves splitting and cloning operations for 3D Gaussian spheres; however, these density control operations are non-differentiable. Since the network gradient must propagate through the rendering process back to each Gaussian parameter and eventually to the neural network parameters, introducing a non-differentiable density control strategy would negatively impact both generalization and novel view rendering accuracy. Consequently, we freeze the adaptive density control process in 3DGS and use alternative regularization strategies to optimize each 3DGS’s parameters.

Our network utilizes an improved version of the Unet^14^ model, known as the Unet++^15^ network, to predict each pixel’s Gaussian sphere attribute parameters. Compared to Unet, Unet++ has the advantage of extracting multi-level features and integrating them through feature stacking, which allows the network to handle different receptive field sizes. Features at different levels exhibit varying sensitivities to objects of different sizes. Additionally, edge information of large objects and details of smaller objects are often lost during repeated downsampling and upsampling in deeper networks. Since UNet++ employs various receptive field sizes, it outperforms Unet in preserving these details. The final layer of the network is replaced with a 1 × 1 convolutional layer with an output channel size of 11 + 3*(J + 1)^2^. We set the order J to 3, resulting in a total of 59 predicted parameters for each Gaussian ellipsoid. These parameters include opacity $\alpha \in {\mathbb{R}}^{+}$, rotation $R\in {\mathbb{R}}^{4}$, scaling $S\in {\mathbb{R}}^{3}$, as well as center offsets in the x directions $\Delta x\in {\mathbb{R}}$, y offsets in the y directions $\Delta y\in {\mathbb{R}}$, and z offsets in the z directions, and color parameters $3*{\left(J+1\right)}^{2}\in {\mathbb{R}}$.The center of the Gaussian ellipsoid is determined based on the depth estimator’s output, with 3D coordinates in view frustum space denoted by

9 $$x,y,z = \left( {\frac{{u_{x} }}{d}f,\;\frac{{u_{y} }}{d}f,d} \right)$$

**Fig. 3:**
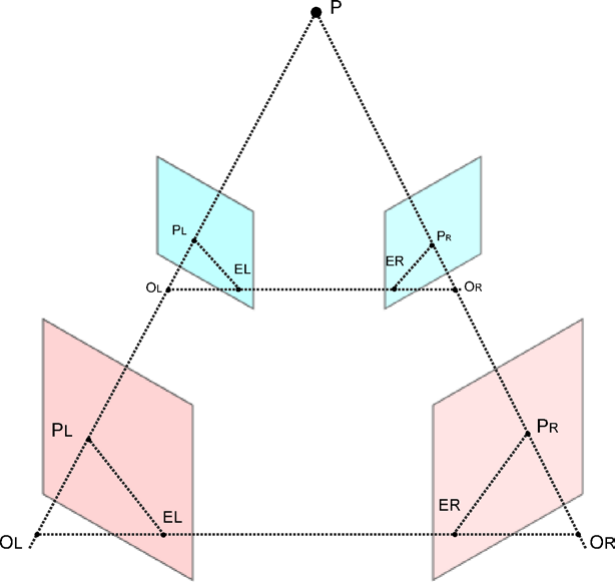
Scale Ambiguity of the SFM Algorithm. As shown in the diagram, due to scale relationships different translation vectors t lead to varying depth calculations for point P thus, the depth values of reconstructed pixels are also dependent on scale.

However, since the depth estimator we use outputs relative depth values, we employed the RealEstate10k dataset for training. This dataset was generated using the SFM algorithm to recover camera motion poses and sparse point cloud data within scenes. As shown in Fig. 3. The SFM algorithm has an inherent scale ambiguity, which arises primarily because the SFM method estimates the rotation matrix (R) and translation vector (t) using eigenvalue decomposition of the essential matrix, leading to an unavoidable uncertainty. Fortunately, once the poses of the first two frames are determined, the SFM algorithm uses the Perspective-n-Point (PnP)^52^ algorithm to estimate the pose transformations between subsequent frames, ensuring consistent scale across all poses within a video sequence.

Our network frontend utilizes a monocular depth estimator to determine the initial positions of each 3D Gaussian ellipsoid in the first layer, resulting in relative depth values. When training the network with consecutive frames, scale consistency issues arise. Thus, the primary objective of network optimization is to resolve scale consistency. Our approach leverages the COLMAP algorithm to perform SFM on image sequences, generating sparse point clouds in 3D space. Once camera poses are obtained, each 3D point is projected from the world coordinate system to the pixel coordinate system according to Eq. (10), identifying the pixel coordinates of each point.

10 $$p=\frac{1}{{z}_{c}}K\left[R|t\right]P$$

where $p\in {\mathbb{R}}^{2}$ is the 2D pixel coordinate in the image, z_c_ is the depth of point *P* in the camera coordinate system $K\in {\mathbb{R}}^{3\times 3}$ is the camera intrinsic matrix, $\left[R|t\right]\in {\mathbb{R}}^{3\times 4}$ is the extrinsic matrix composed of rotation *R* and translation *t*, $P\in {\mathbb{R}}^{3}$ is the 3D point in world coordinates.

The depth estimator network estimates the depth value Z_net_ for each corresponding pixel. Using the specified formula (11), a Scale value is computed for each image.

11 $$Scale=\mathit{exp}\left[\frac{1}{\left|{P}_{spare}\right|}{\sum }_{\left(x,y,z\right)\in {P}_{spare}}\left(\mathit{ln}{Z}_{net}\left(x,y\right)-\mathit{ln}Z\right)\right]$$

where *Scale* is the depth scale alignment factor, *P*_spare_ is the set of sparse depth points obtained via SfM (e.g., COLMAP), ${Z}_{net}\left(x,y\right)$ is the depth value predicted by the monocular depth network at pixel (*x*,*y*), Z is the corresponding ground truth or sparse depth value from SfM, The depth estimation values are adjusted according to the formula:

12 $$depth=\frac{{Z}_{\text{net}}}{S{\text{cale}}}$$

where depth is the final scale-corrected depth value, *Z*net is the predicted depth from the monocular network, *Scale* is the global scale alignment factor computed using sparse SfM points (from Eq. 11). This formula ensuring that the depth scale is consistent with that used in the current dataset. With this scale constraint, the rendered images maintain pixel alignment across synthesized viewpoints, avoiding pixel deviations. Because the neural network predicts the parameters of 3D Gaussians while the density control scheme in the 3D Gaussian rendering process is fixed, the model often overfits to the input viewpoint. To improve the network’s generative capacity for new viewpoints, we incorporate adjacent frames to the target frame during training, constraining the network’s predictions of 3D Gaussian parameters. In prior work, the network first estimated a 3D Gaussian at a central location on the depth surface. Then, it layered predictions of the Gaussian ellipsoid’s position and estimated the directional offset for each layer of the Gaussian. The purpose of this layered prediction approach is to enable the Gaussian model to account for occlusions and support novel viewpoint synthesis. However, this approach has a critical drawback: the generated Gaussian model suffers from poor geometric fidelity. This is largely due to the calculation method, where the depth values are mixed by substituting color with the Gaussian center’s depth in the original rendering formula. The formula for depth information α-blending is

13 $${D}_{a}={\sum }_{i\in N}{d}_{i}{\alpha }_{i}{\prod }_{j=1}^{i-1}\left(1-{\alpha }_{i}\right)$$

where *D*_*a*_ is the final composited depth value of a pixel, *N* is the ordered set of 3D Gaussian indices projected to the pixel (sorted front-to-back by depth), *d*_*i*_ is the depth value (e.g., z-value in camera space) of the *i*-th Gaussian, α_*i*_ ∈ [0,1] α_i_ ∈ [0,1] is the opacity of the *i*-th Gaussian.

**Fig. 4:**
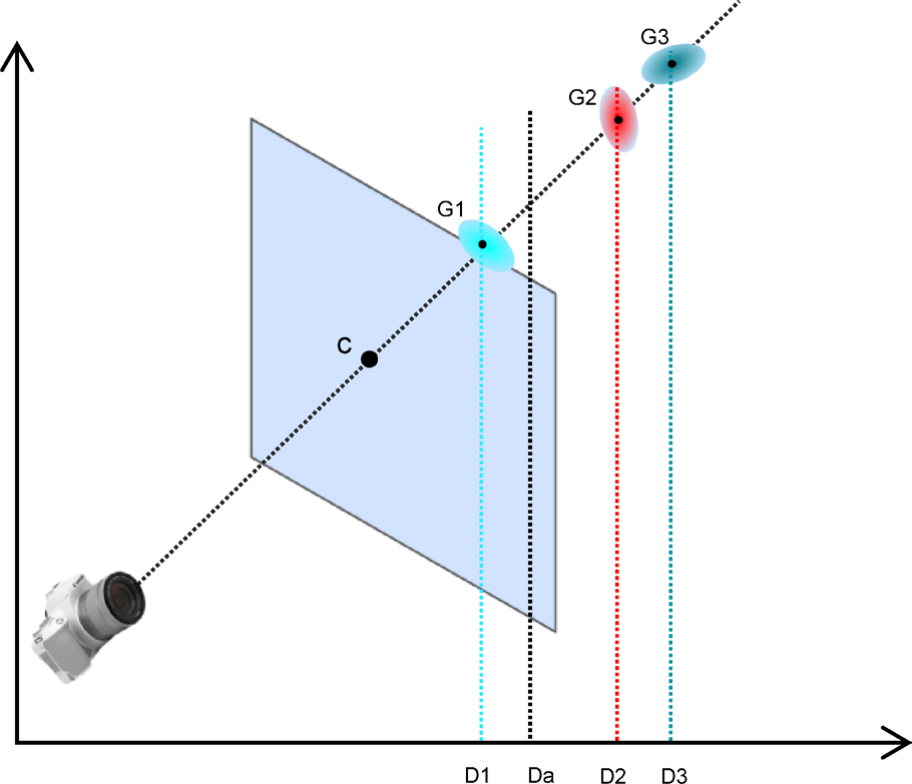
Depth Value Shifting in Multi-layer 3D Gaussian Ellipsoid Rendering. A layered approach for training the neural network is applied, resulting in a 3DGS reconstruction of the image. In this scenario, the network predicts 3D Gaussian properties for pixel c, with depth values D1, D2, and D3. These Gaussian ellipsoids’ parameters ensure correct color rendering when projected onto pixel c. However, when rendering the depth values of the Gaussian ellipsoids G1, G2, and G3, the resultant depth value Da for pixel c differs from the depth value predicted by the depth estimator. This discrepancy, when extended to each pixel, results in a completely disordered geometric rendering for the entire 3DGS model.

As shown in Fig. 4 , according to the 3D Gaussian parameter prediction scheme in flash3D^47^, the final depth value for each pixel is behind the depth surface. Furthermore, the offset amount varies proportionally for each pixel. However, based on prior research, accurate geometric information is essential for synthesizing new viewpoints effectively.

To ensure the network accurately reflects scene geometry while avoiding overfitting on object depth surfaces, we aim to enhance its predictive capacity for unseen regions and its ability to synthesize novel viewpoints. Our network approach involves predicting the coordinates of M Gaussian ellipsoids centers along the ray direction for each pixel based on the camera’s viewpoint. Instead of strictly dividing the Gaussian into multiple layers, we delegate the position adjustments to the neural network, allowing it to determine appropriate offsets. However, this approach risks the 3D Gaussians focusing modeling on the object surface, potentially reducing accuracy and generalization. To address this, we introduce a regularization approach during network training to construct appropriate loss functions, enforcing the network to learn geometric correspondences with adjacent views. Specifically, we input the previously estimated camera transformation matrix and corresponding depth values into the homography transformation^53^ formula

14 $${p}_{t}=K\left(R-\frac{t{n}^{T}}{d}\right){K}^{-1}{p}_{s}$$

where ${p}_{t}\in {\mathbb{R}}^{3}$ is the homogeneous coordinate of a projected pixel in the target view, ${p}_{s}\in {\mathbb{R}}^{3}$ is the homogeneous coordinate of a pixel in the source view, $K\in {\mathbb{R}}^{3\times 3}$ is the intrinsic matrix of the camera, $R\in {\mathbb{R}}^{3\times 3}$ is the relative rotation matrix between the source and target views, $t\in {\mathbb{R}}^{3}$ is the relative translation vector between the two views, $n\in {\mathbb{R}}^{3}$ is the normal vector of the reference plane (usually pointing along the camera’s z-axis in fronto-parallel approximation), $d\in {\mathbb{R}}$ is the depth of the source pixel along the ray (distance to the plane from the camera center).

Using this formula, we compute each pixel’s corresponding position in the adjacent frame. The Gaussian model predicted by the network is then used to perform splatting operations across neighboring views. The purpose of regularization constraints is to ensure depth consistency across both adjacent and target views, achieved through strict geometric constraints. These constraints correct the predicted movement and orientation of Gaussian ellipsoids, preventing overfitting and enhancing the network’s capacity for occlusion prediction and novel view synthesis. In other words, the anchoring effect of the homography transformation helps prevent 3D Gaussian ellipsoids from concentrating excessively on object surfaces, reducing overfitting risks. This also enhances the model’s ability to accurately reconstruct occluded regions and synthesize new viewpoints. The final homography transformation depth loss is therefore:

15 $${L}_{H}=\frac{1}{n}{\sum }_{i=1}^{n}{\left({d}_{i}-{\widehat{d}}_{i}\right)}^{2}$$

where *L*_*H*_ is the homography transformation depth loss, n is the number of valid depth points sampled or matched across views, d_i_ is the predicted depth value of the *i*-th pixel (usually in the target view), ${\widehat{d}}_{i}$ is the reference or warped depth value of the *i*-th pixel, computed by projecting the pixel from the source view to the target view using the homography transform (e.g., from Eq. 14).

Additionally, regarding depth value regularization, as previously mentioned, monocular depth estimation tends to poorly represent geometric shapes, making it unsuitable for direct use in 3D reconstruction. Therefore, we employ 3DGS as a 3D representation method to render 3D scenes. Leveraging the inherent advantage of 3DGS in reconstructing occluded and invisible regions, this approach compensates for the geometric features lost in monocular depth estimation. Directly applying a loss function to each pixel’s estimated depth and rendered depth focuses the network on individual points’ rendering depth without considering the depth of nearby pixels. Any outliers in the depth estimation process will directly affect the overall loss value.

With excessive outliers, regularization may fail, leading to deviations in the final rendering output. Moreover, this regularization approach can somewhat undermine the capability of 3DGS to compensate for geometric losses resulting from depth estimation. We adopted a depth consistency approach to constrain depth loss, effectively addressing the issues encountered in prior methods. Using the Pearson correlation coefficient^54^, we evaluate the correlation in depth value distribution within each patch^55^. This approach means that, rather than comparing two selected points in each iteration, we assess the correlation across an entire patch. By doing so, we can influence a larger area of the image and capture more local structural information. For patch allocation, we assume a patch size of M and refine the patches based on existing configurations. Specifically, we merge patches into sizes of 4 × 4, 8 × 8, and 16 × 16. From each patch size, we randomly select N patches to calculate the depth correlation loss. The final depth loss is obtained by averaging the loss values across all patch configurations. This method enables the network to learn to estimate the parameters of the 3D Gaussian distribution based on depth information and neighboring points. The formula for calculating the Pearson correlation coefficient is:

16 $$PCC\left({d}_{\alpha },{d}_{e}\right)=\frac{E\left({d}_{\alpha }{d}_{e}\right)-E\left({d}_{\alpha }\right)E\left({d}_{e}\right)}{\sqrt{E\left({d}_{\alpha }^{2}\right)-E{\left({d}_{\alpha }\right)}^{2}}\sqrt{E\left({d}_{e}^{2}\right)-E{\left({d}_{e}\right)}^{2}}}$$

where *d*_*a*_ and *d*_*e*_ are the vectors of predicted and estimated depth values (e.g., from the patch), E(⋅) denotes the expectation (mean) operator over all pixels in the patch, PCC(d_a_,d_e_) is the Pearson correlation coefficient between the predicted and estimated depth vectors.

The corresponding loss function is:

17 $${L}_{D}=\frac{1}{N}{\sum }_{i=1}^{n}1-PC{C}_{patch}\left({d}_{\alpha }-{d}_{e}\right)$$

where *L*_*D*_ is the depth correlation loss across all sampled patches, *N* is the number of sampled patches, PCC_patch_(d_a_ − d_e_) is the Pearson correlation of depth difference within each patch (or patch-wise), 1 − *PCC* penalizes negative correlation or uncorrelated depth structures.

The photometric loss in model rendering still uses the L1 loss and SSIM loss from the vanilla 3DGS^9^ approach:

18 $${L}_{c}=\left(1-\lambda \right){L}_{1}+\lambda {L}_{D-SSIM}$$

where *L*_*C*_ is the total photometric loss, L_D-SSIM_ is the structural similarity (SSIM)-based photometric loss, λ ∈ [0,1] is a hyperparameter that balances between color accuracy and structural similarity. when constrained by the photometric loss function, ensures consistent color rendering across different views during splatting. Combining the above three loss functions, the total loss function of the network is:

19 $${L}_{ALL}={L}_{C}+{\beta }_{D}{L}_{D}+{\beta }_{H}{L}_{H}$$

where *L*_ALL_ is the total training loss of the network, L_C_ is the photometric rendering loss (Eq. 18), L_D_ is the depth correlation loss (Eq. 17), L_H_ is the homography-based depth consistency loss (Eq. 15), β_D_ and β_*H*_ are weighting hyperparameters for their respective losses.

## Experiments

We trained and evaluated our network on the large-scale RealEstate10K^16^ dataset, conducting 60,000 iterations on a single 4090 GPU with a batch size of 8 over a period of three days. The RealEstate10K dataset is a large-scale camera pose dataset containing approximately 10 million frames from around 80,000 video clips sourced from 10,000 YouTube videos. These frame sequences are divided into training and testing sets, with each sequence containing the associated camera’s intrinsic and extrinsic parameters. Since consistency between the depth estimation scale and SFM scale is required, we used COLMAP to perform SFM on each video sequence, generating sparse point cloud data. To assess the performance of novel view synthesis, we compared our network with two state-of-the-art methods: MINE, an optimal method for novel view synthesis based on MPI, and Flash3D, an optimal single-view 3D reconstruction approach using 3DGS. To evaluate the network’s generalization ability, we tested it not only on the RealEstate10K test set but also on the KITTI^17^ and NYU^18^ datasets.

### View synthesis on RealEstate10K

We extracted images from various scenes in the validation set of the RealEstate10K dataset. From dozens of images per scene, we selected images at different frame intervals to validate the network’s novel view synthesis capabilities under various baselines. We evaluated the model’s reconstruction quality by measuring the synthesized views’ quality, reporting peak signal-to-noise ratio (PSNR)^56^, structural similarity (SSIM)^57^, and learned perceptual image patch similarity (LPIPS)^56^. For LPIPS computation, we used a VGG16^58^ model pre-trained on the ImageNet dataset.

We define the perspective input into the network for 3D model prediction as the target view and refer to the view of the perspective to be predicted as the source view. In the experiments, we first predict the 3D model from the target view. The predicted model is then rendered from the source view’s perspective and compared with the ground truth to calculate various quantitative analysis metrics. The results in Table 1 show that our model outperforms others in quantitative metrics by rendering and comparing images across various baselines. This improvement is largely due to our model’s regularization strategy, which enables the network to predict the positions of Gaussian ellipsoids and other parameters based on neighboring pixel data.

**Table 1:** Quantitative comparison experiments with other models on the RealEstate10K dataset.

Method	5 Frames			10 Frames			20 Frames			30 Frames		
	PSNR ↑	SSIM↑	LPIPS↓	PSNR↑	SSIM↑	LPIPS↓	PSNR↑	SSIM↑	LPIPS↓	PSNR↑	SSIM↑	LPIPS↓
MPI	26.4	0.859	0.120	23.5	0.795	0.155	22.7	0.706	0.176	21.6	0.693	0.180
MINE	28.43	0.892	0.111	25.85	0.848	0.148	24.88	0.853	0.170	24.74	0.818	0.179
Flash3D	28.46	0.898	0.100	25.93	0.857	0.132	24.96	0.856	0.168	24.93	0.832	1.161
SVG3D	28.49	0.901	0.099	25.95	0.859	0.129	24.98	0.858	0.160	24.94	0.836	0.159

**Fig. 5:**
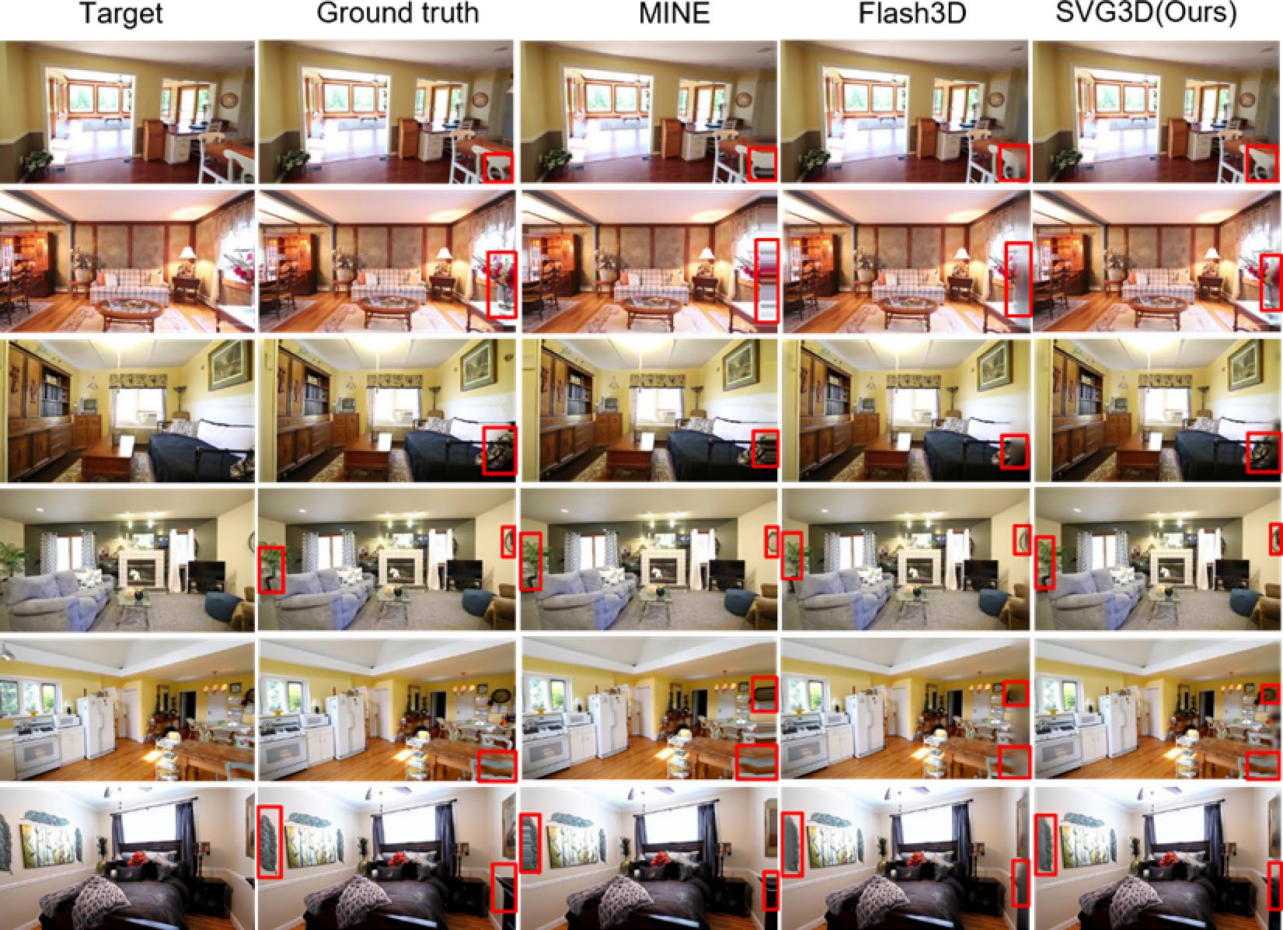
Comparative Experiments on the RealEstate10K Dataset. We conducted comparative experiments with the state-of-the-art single-view 3D reconstruction models, including the hybrid representation of NeRF and MPI-based model (MINE) and the 3DGS-based model (Flash3D). Images from six distinct scenes were selected at intervals of 5 frames. The results clearly demonstrate that our model significantly outperforms the other models in reconstructing novel views.

From the experimental results in Fig. 5, it can be seen that our model, SVG3D, performs exceptionally well in both scene reconstruction and novel view synthesis for target scenes. For instance, in the second scene, the reconstruction of the vase on the right side clearly outperforms the current state-of-the-art method, Flash3D, in both reconstruction quality and color fidelity. Additionally, the reconstruction of the potted plant on the left side in the fourth scene showcases the benefits of the 3DGS technique, an effective hybrid of implicit and explicit 3D representation. This allows the network to accurately restore and infer the details of plant leaves. Overall, SVG3D demonstrates strong performance on the RealEstate10K dataset, achieving impressive results.

### Experiment on network generalization performance

To validate the generalization performance of the network model, we evaluated the model on the KITTI and NYU datasets. During the experiments, we compared the model with the state-of-the-art single-view reconstruction methods: MINE, based on neural radiance fields (NeRF), and Flash3D, based on 3DGS. We conducted both quantitative and qualitative experiments. Since the KITTI and NYU datasets provide scale information for the entire dataset, no additional scale-consistency calculations were necessary for these datasets. Instead, we directly aligned the scale information from the monocular depth estimator with the dataset scale information.

We compare the state-of-the-art monocular 3D reconstruction methods in Table 2, MINE and Flash3D. For quantitative evaluation, we report standard image quality metrics, including pixel-level PSNR, patch-level SSIM, and feature-level LPIPS. The results indicate that our network model performs well even on unseen datasets, consistently surpassing previous models across all metrics.

**Table 2:** Quantitative analysis of cross-dataset generalization experiments.

Method	KITTI			NYU		
	PSNR↑	SSIM↑	LPIPS↓	PSNR↑	SSIM↑	LPIPS↓
MINE	21.62	0.812	0.153	23.86	0.738	0.212
Flash3D	21.80	0.820	0.143	25.10	0.756	0.197
SVG3D(Ours)	21.98	0.830	0.130	25.48	0.780	0.193

**Fig. 6:**
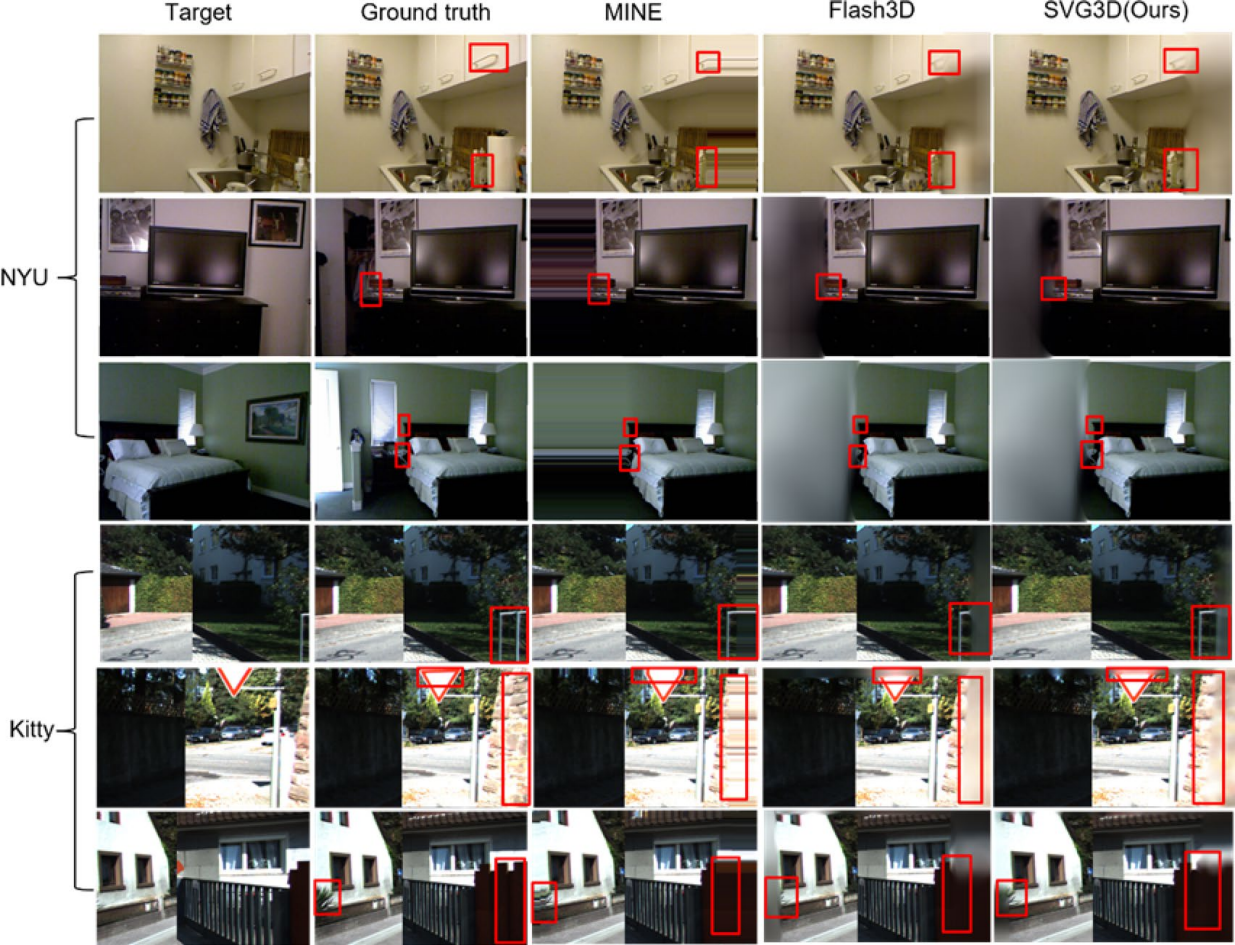
Cross-Dataset Generalization Experiment. Although the images in the KITTI and NYU test sets have a larger baseline comparison, our network demonstrates strong reconstruction capabilities for target viewpoints and performs well in synthesizing new perspectives. We conducted experiments on three distinct scenes from the NYU and KITTI datasets, respectively. The experimental results indicate that SVG3D outperforms other network models.

From the experimental results shown in Fig. 6, it can be observed that our network exhibits robust performance on previously unseen datasets, demonstrating strong cross-dataset generalization. For instance, in the first scene of the NYU dataset, our network accurately reconstructs the occluded areas, such as the upper cabinet handles and lower bottles. Additionally, in the second scene of the KITTI dataset, our model performs well in reconstructing the traffic signs above and the wall sections on the right.

### Network transferability and generalization evaluation

To further evaluate the generalization ability and transfer learning capability of our network, we finetune it on the DTU dataset^16^ and conduct testing on Real Forward-Facing^7^ and Tanks and Temples datasets^59^. These datasets are significantly different in terms of scene structure, lighting conditions, and camera trajectories, making them suitable for evaluating the robustness of the learned 3D representations.

The performance on these unseen datasets demonstrates that our model can effectively adapt to new scenes without requiring retraining from scratch, thus verifying its strong transferability across domains. The quantitative results are shown in Table 3.

**Table 3:** Evaluation of transferability after finetuning on DTU.

Finetuned on	Tested on	PSNR↑	SSIM↑	LPIPS↓
DTU	DTU	27.20	0.951	0.082
DTU	Real forward-facing	23.07	0.812	0.163
DTU	Tanks and temples	22.19	0.793	0.172

This table summarizes the quantitative evaluation of our network’s generalization and transfer learning capability. The model is finetuned on the DTU dataset and then tested on three different datasets: DTU (in-domain), Real Forward-Facing, and Tanks and Temples (both cross-domain). Results show a consistent performance drop when tested on out-of-distribution datasets, which is expected, but the degradation remains moderate, indicating that our model possesses reasonable transferability to unseen domains.

To further verify the generalization ability of our model, we conduct qualitative comparisons on the DTU, Real Forward-Facing, and Tanks and Temples datasets. These visualizations demonstrate how well the model transfers to unseen domains in terms of rendering fidelity and structural consistency.

**Fig. 7:**
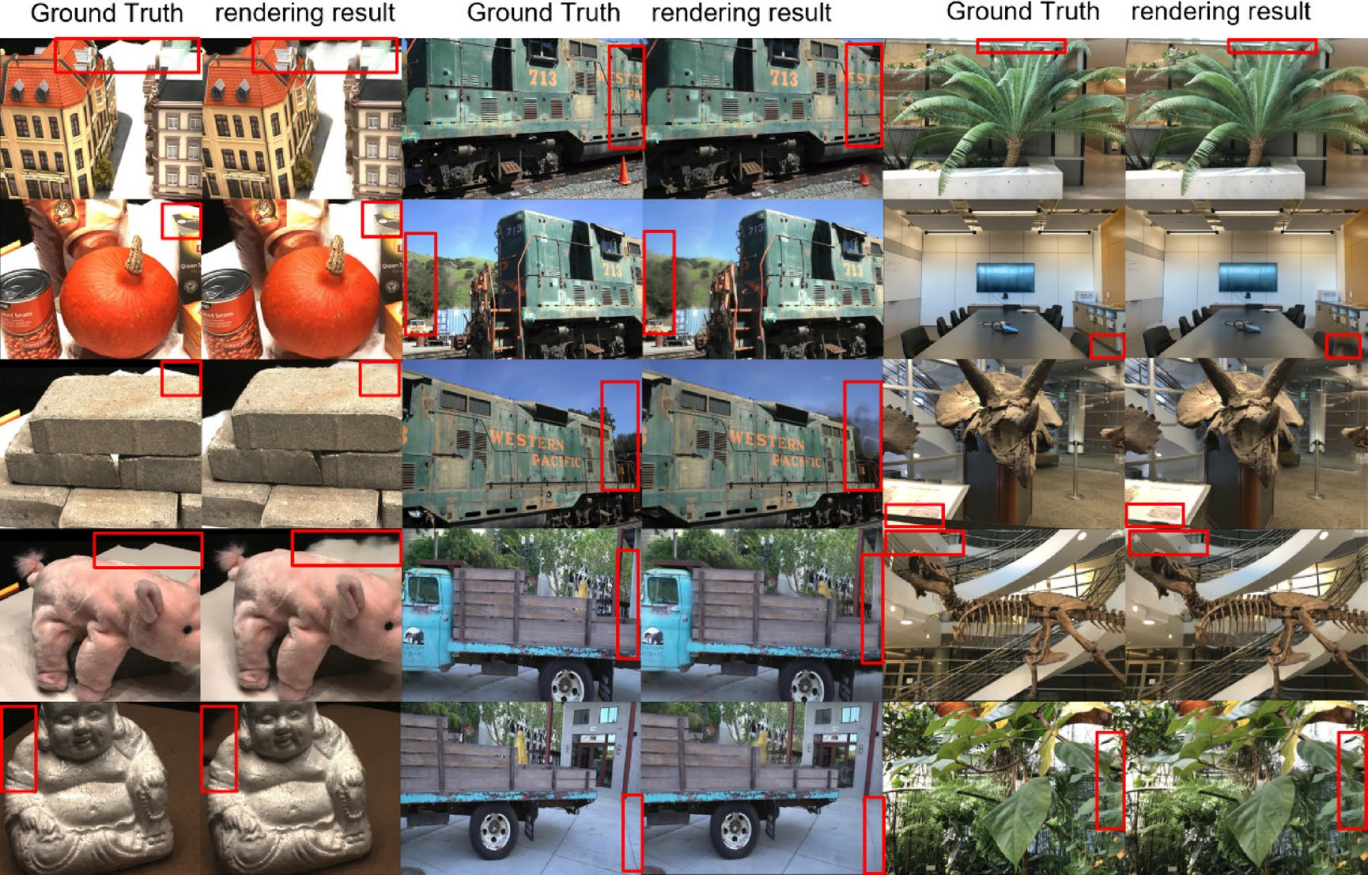
Qualitative comparisons on DTU, Real Forward-Facing, and Tanks and Temples datasets. In each pair, the left image is the ground truth and the right image is the rendering result from our model finetuned on the DTU dataset. Red boxes highlight areas with noticeable reconstruction details and differences. Despite being trained only on DTU, our method shows strong generalization across domains, producing visually faithful results even on unseen datasets.

As illustrated in Fig. 7, we compare the ground truth images (left) with our reconstructed images (right) across three datasets: DTU (in-domain), Real Forward-Facing, and Tanks and Temples (out-of-domain). The results show that our model is capable of maintaining structural consistency and photorealistic rendering quality even in unseen domains, validating its strong transfer learning capability.

Although our method generalizes well to unseen datasets, as demonstrated qualitatively in Fig. 7, it still exhibits certain limitations inherent to single-view 3D reconstruction.

Compared to multi-view systems, our model lacks explicit geometric constraints and depth triangulation, which may result in shape ambiguities, incomplete structures, or texture inconsistencies, particularly in regions with occlusions, thin structures, or reflective materials.

In Fig. 7, several typical failure cases can be observed: In row 2, the pumpkin’s outline becomes blurred and its surface loses fine-grained shading detail, likely due to the lack of 3D cues from multiple viewpoints. In row 4, the pink plush toy exhibits shape distortion around the ears and back, where self-occlusion and soft material deformation present significant challenges. In row 5, the Buddha statue’s edge fidelity is degraded, and subtle reflectance changes are not faithfully reconstructed. the truck model shows geometry misalignment and ghosting near thin structures such as railings and truck sides. In summary, while our method performs well in a wide range of scenarios, addressing the challenges of occlusion reasoning and fine structure recovery remains an open problem for single-view 3D reconstruction.

To better demonstrate the structure and interpretability of the learned scene representation, we visualize the internal 3D Gaussians estimated by our model after training. Each Gaussian encodes position, scale, orientation, color, and opacity, forming a compact and sparse volumetric representation of the scene. We export these learned 3D Gaussian models to ply files and render them using SuperSplat, a rasterizer specifically designed for visualizing Gaussian splats with ellipsoidal shape and opacity cues. This allows us to directly inspect the spatial distribution and geometry of the predicted Gaussians, including their anisotropic contours and density patterns.

**Fig. 8:**
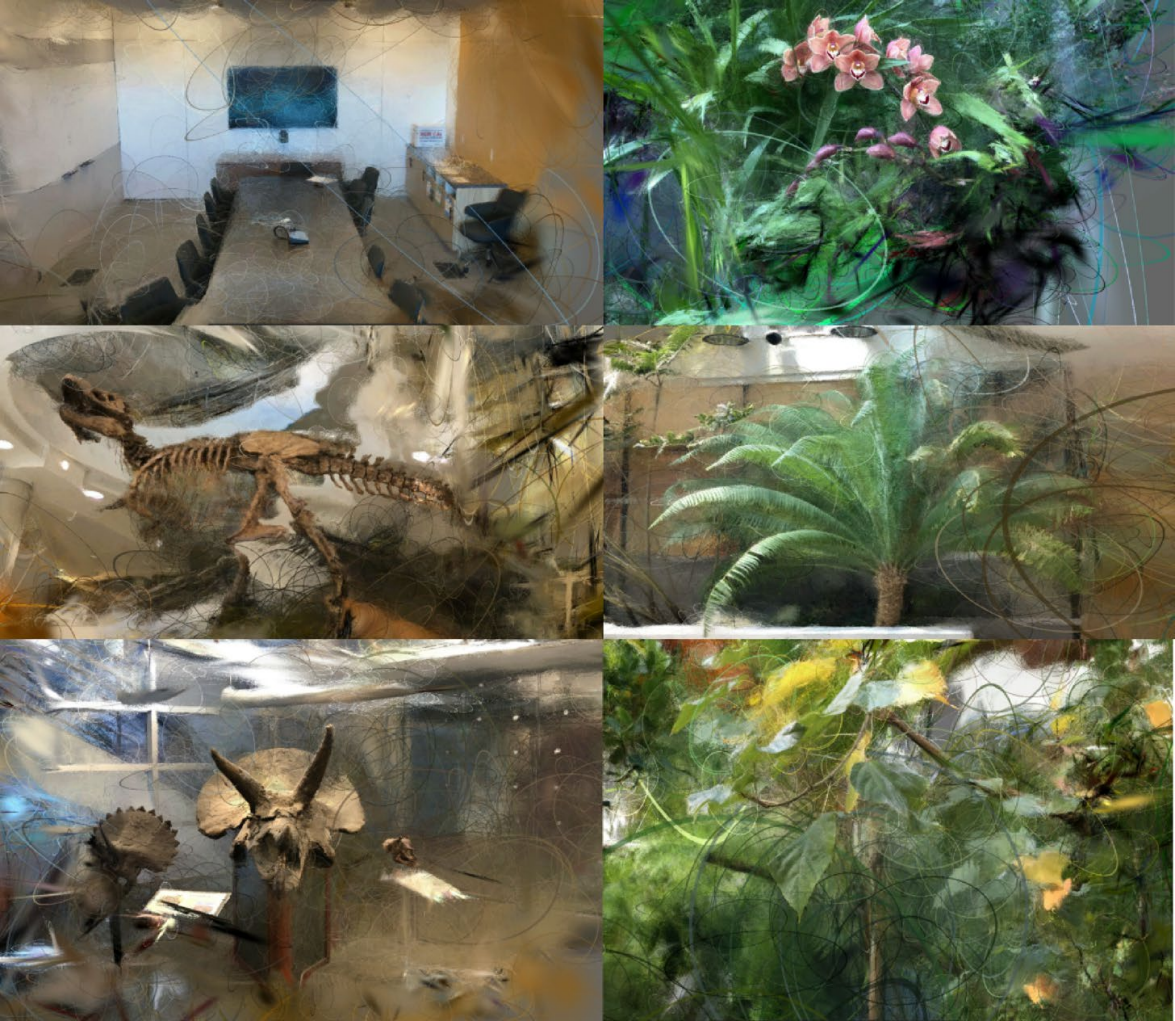
Visualization of learned 3D Gaussians on six scenes from the Real Forward-Facing dataset. Gaussians are exported as ply files and rendered using SuperSplat with ellipsoid and opacity visualization enabled. The learned distributions reveal spatial structure, object boundaries, and feature continuity.

Figure 8 shows visualization results on six representative scenes from the Real Forward-Facing dataset, covering a range of indoor and outdoor environments. The rendered Gaussians form coherent and continuous structures, aligning well with object boundaries and scene layouts. In particular, fine-scale details such as vegetation, fossils, and architectural edges are well captured by the ellipsoidal splats. These results qualitatively confirm that our method produces interpretable and structured scene representations, effectively modeling geometry and appearance in a unified Gaussian-based formulation.

### Runtime efficiency analysis

To further evaluate the algorithmic complexity and practical usability of our method, we conducted a runtime efficiency comparison with two representative single-view 3D reconstruction baselines: Flash3D and MINE.

We compare four metrics: average inference time per frame, training time per 1000 iterations, and peak GPU memory usage. All experiments were conducted on an NVIDIA RTX 4090 GPU (24 GB), with input resolution set to 640 × 384 and batch size = 1. To ensure a fair and consistent comparison, we reproduced both Flash3D and MINE using their official open-source implementations, and evaluated them under identical hardware conditions. It is worth noting that in their original training setups, Flash3D was trained on a single NVIDIA A6000 GPU for 40,000 iterations in approximately 16 h, as reported in their paper. MINE, although lacking detailed runtime information in its original paper, was reproduced by Flash3D’s authors using two A6000 GPUs for 350,000 steps, requiring about 6 GPU-days, according to their supplementary material. By re-implementing and benchmarking both methods on a single RTX 4090 GPU, we are able to provide consistent and directly comparable runtime measurements under real-world conditions.

As summarized in Table 4, SVG3D achieves the fastest inference speed (85.3 ms) and lowest training cost, while maintaining moderate GPU memory usage and compact model size. These results indicate that SVG3D provides a favorable trade-off between quality and efficiency, and demonstrates its potential for practical deployment in 3D vision applications.

**Table 4:** Runtime efficiency comparison.

Method	Inference time (ms)	Train time per 1000 iters (s)	GPU memory usage (MB)
SVG3D (ours)	85.3	410	7100
Flash3D (reproduced)	97.8	460	6700
MINE (reproduced)	152.6	660	8100

## Ablation study

We conducted ablation experiments on the RealEstate10K dataset to analyze the role of various network modules. This analysis addresses and seeks to resolve the following questions:

- Question 1: Is the monocular depth estimator essential within the network model? If we remove the monocular depth estimator and instead initialize the center coordinates of the 3D Gaussian ellipsoid with a random number, can the model still accurately predict the 3D representation of a single view and synthesize new viewpoints?
- Question 2: Is the regularization loss for the depth values at corresponding points in the homography matrix necessary? What role does this depth regularization loss serve in the network?
- Question 3: During the reconstruction of the target view, we compare the depth value predicted by the monocular depth map to the depth obtained after α-blending with the 3D Gaussian model. Rather than calculating depth loss by comparing individual pixel depths at each iteration, the network instead compares pixels within an entire patch, using different patch sizes to compute the Pearson correlation coefficient. Is this approach necessary, and what advantages does it offer over directly comparing pixel depth losses?

To address the above questions, we isolated each component of the network and conducted experiments on the RealEstate10K dataset. The experimental results are shown in Table 5, with Rows 2 through 4 corresponding to Experiments 1 to 3, respectively.

**Table 5:** Ablation study of different modules in the network model.

Method	PSNR↑	SSIM↑	LPIPS↓
SVG3D (All modules are available)	28.49	0.901	0.099
No monocular depth estimator	18.63	0.602	0.321
No LH regularization loss	21.47	0.823	0.158
No Pearson correlation coefficient regularization loss	23.56	0.836	0.150

From the Table 5's quantitative results of the ablation study, it is clear that the monocular depth estimation module, homography-based depth regularization module, and Pearson correlation depth regularization module are indispensable. The quantitative data in Row 2 indicates that, without initializing the center coordinates of the 3D Gaussian ellipsoid through monocular depth estimation, model performance declines significantly. This decline primarily stems from the model’s lack of supplementary information due to reliance on a single view. Randomly initialized 3D Gaussian ellipsoids cannot accurately represent scene geometry, and the network cannot autonomously adjust their positions during training, leading to substantial performance degradation. This highlights the importance of monocular depth estimation in providing precise geometric constraints to guide the network in initializing the 3D Gaussian ellipsoid’s center coordinates.

The quantitative data in Row 3 shows that the network’s performance is also heavily impacted in the absence of homography regularization. To ensure the predicted 3D Gaussian ellipsoid distributions accurately reflect depth characteristics, our model does not impose rigid layering on the ellipsoids. Instead, the network adjusts their positions to maintain accurate α-blending depth values. However, this approach can lead to overfitting on the target view, with 3D Gaussian ellipsoids clustering on object surfaces, which undermines the network’s ability to synthesize novel viewpoints. Thus, homography regularization serves as a geometric anchor that prevents ellipsoids from overfitting on object surfaces, while ensuring occlusion region reconstruction and enhancing the accuracy of novel viewpoint synthesis.

Row 4’s quantitative analysis highlights the significance of the Pearson correlation coefficient loss function. Solely applying an L2 loss on pixel depth values would ultimately degrade model performance. This issue arises primarily due to inevitable outliers in monocular depth estimation, which can distort loss computation. Additionally, using patches of varying sizes to calculate the Pearson correlation enables the network to integrate global and local depth information for more accurate 3D Gaussian ellipsoid positioning, thereby improving network precision.

## Conclusion

We propose a 3D reconstruction model based on 3DGS, which can generate a 3D reconstruction from a single input view and synthesize novel perspectives of the scene. To achieve such a challenging task from a single image, we use an advanced monocular depth estimation model (DepthAnythingV2) to predict the depth value for each pixel in the scene. These depth predictions are then input into the neural network to provide additional constraint information. During network training, we designed a depth regularization loss based on homography transformation for adjacent views, along with a depth estimation regularization loss based on the Pearson correlation coefficient for target views. These losses help the network achieve effective occlusion handling and novel view synthesis. We trained and validated our model on the RealEstate10K dataset, achieving satisfactory results. To further test the network’s generalization capabilities, we conducted quantitative and qualitative evaluations on the KITTI and NYU datasets, where our model outperformed other single-view 3D reconstruction models.

Despite the promising results, our method still has several limitations. First, as a single-view reconstruction system, the model naturally suffers from depth ambiguity and occlusion uncertainty, especially in scenes with complex geometry or weak textures. Although we incorporate monocular depth estimation and novel loss functions to mitigate these issues, the lack of multi-view constraints limits the precision of geometry recovery in fine structures or occluded areas. In addition, the reliance on a pretrained depth estimator may introduce domain bias when applied to scenes that differ significantly from the training distribution.

In future work, we plan to explore the integration of learnable geometric priors or multi-task supervision (e.g., surface normal or semantic segmentation) to improve depth consistency and robustness. We also consider introducing lightweight multi-view refinement modules during inference to enhance reconstruction quality without requiring full multi-view input during training. These directions could further close the performance gap between single-view and multi-view 3D reconstruction methods.

## Data Availability

The datasets used and/or analysed during the current study available from the corresponding author on reasonable request.

## References

[1] Poole, B., Jain, A., Barron, J. T. & Mildenhall, B. Dreamfusion: Text-to-3d using 2d diffusion. *arXiv preprint *arXiv:2209.14988 (2022).

[2] Lin, C.-H. *et al.* in *Proceedings of the IEEE/CVF conference on computer vision and pattern recognition.* 300–309.

[3] Deng, C. *et al.* in *Proceedings of the IEEE/CVF conference on computer vision and pattern recognition.* 20637–20647.

[4] Nichol, A., Jun, H., Dhariwal, P., Mishkin, P. & Chen, M. Point-e: A system for generating 3d point clouds from complex prompts. *arXiv preprint *arXiv:2212.08751 (2022).

[5] Chan, E. R. *et al.* in *Proceedings of the IEEE/CVF international conference on computer vision.* 4217–4229.

[6] Xu, D. *et al.* in *Proceedings of the IEEE/CVF conference on computer vision and pattern recognition.* 4479–4489.

[7] Mildenhall, B. et al. Nerf: Representing scenes as neural radiance fields for view synthesis. *Commun. ACM* **65**, 99–106 (2021).

[8] Park, J. J., Florence, P., Straub, J., Newcombe, R. & Lovegrove, S. in *Proceedings of the IEEE/CVF conference on computer vision and pattern recognition.* 165–174.

[9] Kerbl, B., Kopanas, G., Leimkühler, T. & Drettakis, G. 3D Gaussian splatting for real-time radiance field rendering. *ACM Trans. Graph.* **42**, 139:131–139:114 (2023).

[10] Yu, A., Ye, V., Tancik, M. & Kanazawa, A. in *Proceedings of the IEEE/CVF conference on computer vision and pattern recognition.* 4578–4587.

[11] Yin, W., Liu, Y., Shen, C. & Yan, Y. in *Proceedings of the IEEE/CVF international conference on computer vision.* 5684–5693.

[12] Yin, W. *et al.* in *Proceedings of the IEEE/CVF conference on computer vision and pattern recognition.* 204–213.

[13] Yin, W. *et al.* in *Proceedings of the IEEE/CVF international conference on computer vision.* 9043–9053.

[14] Ronneberger, O., Fischer, P. & Brox, T. in *Medical image computing and computer-assisted intervention–MICCAI 2015: 18th international conference, Munich, Germany, October 5–9, 2015, proceedings, part III 18.* 234–241 (Springer).

[15] Zhou, Z., Rahman Siddiquee, M. M., Tajbakhsh, N. & Liang, J. in *Deep learning in medical image analysis and multimodal learning for clinical decision support: 4th International workshop, DLMIA 2018, and 8th international workshop, ML-CDS 2018, held in conjunction with MICCAI 2018, Granada, Spain, September 20, 2018, Proceedings 4.* 3–11 (Springer). 10.1007/978-3-030-00889-5_1 PMC7329239 32613207

[16] Zhou, T., Tucker, R., Flynn, J., Fyffe, G. & Snavely, N. Stereo magnification: Learning view synthesis using multiplane images. *arXiv preprint *arXiv:1805.09817 (2018).

[17] Geiger, A., Lenz, P. & Urtasun, R. in *2012 IEEE conference on computer vision and pattern recognition.* 3354–3361 (IEEE).

[18] Silberman, N., Hoiem, D., Kohli, P. & Fergus, R. in *Computer Vision–ECCV 2012: 12th European conference on computer vision, Florence, Italy, October 7–13, 2012, Proceedings, Part V 12.* 746–760 (Springer).

[19] Li, J. *et al.* in *Proceedings of the IEEE/CVF international conference on computer vision.* 12578–12588.

[20] Riegler, G. & Koltun, V. in *Computer vision–ECCV 2020: 16th European conference, Glasgow, UK, August 23–28, 2020, Proceedings, Part XIX 16.* 623–640 (Springer).

[21] Tucker, R. & Snavely, N. in *Proceedings of the IEEE/CVF conference on computer vision and pattern recognition.* 551–560.

[22] Wizadwongsa, S., Phongthawee, P., Yenphraphai, J. & Suwajanakorn, S. in *Proceedings of the IEEE/CVF conference on computer vision and pattern recognition.* 8534–8543.

[23] Gao, J. et al. Get3d: A generative model of high quality 3d textured shapes learned from images. *Adv. Neural. Inf. Process. Syst.* **35**, 31841–31854 (2022).

[24] Liu, M. et al. One-2-3-45: Any single image to 3d mesh in 45 seconds without per-shape optimization. *Adv. Neural. Inf. Process. Syst.* **36**, 22226–22246 (2023).

[25] Liu, R. *et al.* in *Proceedings of the IEEE/CVF international conference on computer vision.* 9298–9309.

[26] Müller, N. *et al.* in *Proceedings of the IEEE/CVF conference on computer vision and pattern recognition.* 4328–4338.

[27] Chen, H. *et al.* in *Proceedings of the IEEE/CVF international conference on computer vision.* 2416–2425.

[28] Shi, R., Wei, X., Wang, C. & Su, H. in *Proceedings of the IEEE/CVF conference on computer vision and pattern recognition.* 21114–21124.

[29] Hong, Y. *et al.* Lrm: Large reconstruction model for single image to 3d. *arXiv preprint *arXiv:2311.04400 (2023).

[30] Chen, Y. *et al.* Mvsplat360: Feed-forward 360 scene synthesis from sparse views. *arXiv preprint *arXiv:2411.04924 (2024).

[31] Chen, A. *et al.* in *Proceedings of the IEEE/CVF international conference on computer vision.* 14124–14133.

[32] Debevec, P. E., Taylor, C. J. & Malik, J. in *Seminal graphics papers: Pushing the boundaries**, **volume 2* 465–474 (2023).

[33] Radford, A. *et al.* in *International conference on machine learning.* 8748–8763 (PMLR).

[34] Alexey, D. An image is worth 16x16 words: Transformers for image recognition at scale. *arXiv preprint *arXiv: 2010.11929 (2020).

[35] Metzer, G., Richardson, E., Patashnik, O., Giryes, R. & Cohen-Or, D. in *Proceedings of the IEEE/CVF conference on computer vision and pattern recognition.* 12663–12673.

[36] Wang, C. *et al.* Nerf-art: Text-driven neural radiance fields stylization. *IEEE Trans. Visual. Comput. Graph.* (2023). 10.1109/TVCG.2023.3283400 37279137

[37] Zhang, J., Li, X., Wan, Z., Wang, C. & Liao, J. Text2nerf: Text-driven 3d scene generation with neural radiance fields. *IEEE Trans. Visual. Comput. Graph.* (2024). 10.1109/TVCG.2024.3361502 38315587

[38] Yang, J., Pavone, M. & Wang, Y. in *Proceedings of the IEEE/CVF conference on computer vision and pattern recognition.* 8254–8263.

[39] Niemeyer, M. *et al.* in *Proceedings of the IEEE/CVF conference on computer vision and pattern recognition.* 5480–5490.

[40] Charatan, D., Li, S. L., Tagliasacchi, A. & Sitzmann, V. in *Proceedings of the IEEE/CVF conference on computer vision and pattern recognition.* 19457–19467.

[41] Chen, Y. *et al.* in *European conference on computer vision.* 370–386 (Springer).

[42] Xiong, H. *Sparsegs: Real-time 360° sparse view synthesis using gaussian splatting* (University of California, 2024).

[43] Li, J. *et al.* in *Proceedings of the IEEE/CVF conference on computer vision and pattern recognition.* 20775–20785.

[44] Yu, H., Long, X. & Tan, P. LM-Gaussian: Boost Sparse-view 3D Gaussian splatting with large model priors. *arXiv preprint *arXiv:2409.03456 (2024).

[45] Wang, S., Leroy, V., Cabon, Y., Chidlovskii, B. & Revaud, J. in *Proceedings of the IEEE/CVF conference on computer vision and pattern recognition.* 20697–20709.

[46] Szymanowicz, S., Rupprecht, C. & Vedaldi, A. in *Proceedings of the IEEE/CVF conference on computer vision and pattern recognition.* 10208–10217.

[47] Szymanowicz, S. *et al.* Flash3D: Feed-forward generalisable 3D scene reconstruction from a single image. *arXiv preprint *arXiv:2406.04343 (2024).

[48] Schonberger, J. L. & Frahm, J.-M. in *Proceedings of the IEEE conference on computer vision and pattern recognition.* 4104–4113.

[49] Zwicker, M., Pfister, H., Van Baar, J. & Gross, M. in *Proceedings visualization, 2001. VIS’01.* 29–538 (IEEE).

[50] Yang, L. *et al.* in *Proceedings of the IEEE/CVF conference on computer vision and pattern recognition.* 10371–10381.

[51] Yang, L. *et al.* Depth Anything V2. *arXiv preprint *arXiv:2406.09414 (2024).

[52] Lowe, D. G. Fitting parameterized three-dimensional models to images. *IEEE Trans. Pattern Anal. Mach. Intell.* **13**, 441–450 (1991).

[53] Hartley, R. & Zisserman, A. *Multiple view geometry in computer vision*. (Cambridge university press, 2003).

[54] Pearson, K. VII. Mathematical contributions to the theory of evolution.—III. Regression, heredity, and panmixia. *Philos. Trans. R. Soc. Lond. Ser. A Contain. Papers of a mathematical or physical character*, 253–318 (1896).

[55] Liu, Z. *et al.* in *Proceedings of the IEEE/CVF international conference on computer vision.* 10012–10022.

[56] Zhang, R., Isola, P., Efros, A. A., Shechtman, E. & Wang, O. in *Proceedings of the IEEE conference on computer vision and pattern recognition.* 586–595.

[57] Wang, Z., Bovik, A. C., Sheikh, H. R. & Simoncelli, E. P. Image quality assessment: From error visibility to structural similarity. *IEEE Trans. Image Process.* **13**, 600–612 (2004). 15376593 10.1109/tip.2003.819861

[58] Simonyan, K. Very deep convolutional networks for large-scale image recognition. *arXiv preprint *arXiv:1409.1556 (2014).

[59] Knapitsch, A., Park, J., Zhou, Q.-Y. & Koltun, V. Tanks and temples: Benchmarking large-scale scene reconstruction. *ACM Trans. Graph.* **36**, 1–13 (2017).

